# Social and spatial patterns of obesity diffusion over three decades in a Norwegian county population: the HUNT Study

**DOI:** 10.1186/1471-2458-13-973

**Published:** 2013-10-19

**Authors:** Steinar Krokstad, Linda Ernstsen, Erik R Sund, Johan Håkon Bjørngaard, Arnulf Langhammer, Kristian Midthjell, Turid Lingaas Holmen, Jostein Holmen, Håvard Thoen, Steinar Westin

**Affiliations:** 1 HUNT Research Centre, Department of Public Health and General Practice, Faculty of Medicine, Norwegian University of Science and Technology, Levanger, Norway; 2 Levanger Hospital, Nord-Trøndelag Health Trust, Levanger, Norway; 3 Faculty of Nursing Education, Sør-Trøndelag University College, Trondheim, Norway; 4 Department of Public Health and General Practice, Faculty of Medicine, Norwegian University of Science and Technology, Trondheim, Norway; 5 Statistics Norway, Oslo, Norway

## Abstract

**Background:**

In order to develop effective preventive strategies, knowledge of trends in socioeconomic and geographical differences in risk factor levels is important. The objective of this study was to examine social and spatial patterns of obesity diffusion in a Norwegian population during three decades.

**Methods:**

Data on adults aged 30–69 years from three cross-sectional health surveys eleven years apart in the Nord-Trøndelag Health Study, Norway, HUNT1 (1984–1986), HUNT2 (1995–1997) and HUNT3 (2006–2008) were utilized. Body mass index (BMI) was used as a measure of obesity. Height and weight were measured clinically. Age standardized prevalences, absolute prevalence differences and ratios, prevalence odds ratios for BMI and the Relative Index of Inequality (RII) were calculated. Multilevel statistical models were fitted for analysing geographical patterns.

**Results:**

The prevalence of obesity was systematically higher in groups with lower socio-economic status and increased successively in all groups in the population during the three decades. The relative socioeconomic inequalities in obesity measured by level of education did not change substantially in the period. In HUNT1 (1984–86) obesity was most prevalent among low educated women (14.1%) and in HUNT3 (2006–08) among low educated men (30.4%). The RII for men changed from 2.60 to 1.91 and 2.36 in HUNT1, HUNT2 and HUNT3. In women the RIIs were 1.71, 2.28 and 2.30 correspondingly. However, the absolute obesity prevalence inequalities increased, and a geographical diffusion from central to distal districts was observed from HUNT2 to HUNT3.

**Conclusions:**

The prevalence of obesity increased in all socioeconomic groups in this Norwegian adult county population from the 1980ies up to present time. The data did not suggest increasing relative inequalities, but increasing absolute socioeconomic differences and a geographical diffusion towards rural districts. Public health preventive strategies should be oriented to counteract the obesity epidemic in the population.

## Background

The prevalence of obesity is known to increase not only in industrialized countries [1,2], but also in developing countries, when traditional living conditions are changed by influence from developed economies [3-5]. Although economic and social development can improve health, it can also lead to increasing obesity and widening socioeconomic disparities in obesity [6]. Numerous studies have addressed socioeconomic inequalities in obesity, and suggested that persons with high socioeconomic status (SES) tend to weigh more than others in poor countries whereas an opposite pattern is found in rich countries [7]. Thus, the mechanisms behind this development are probably related to changes in health related behaviour associated with social and economic development [8].

In a review article from 2005, Ball and Crawford studied the relationship of SES to weight change over time in several developed countries, reporting relatively consistent inverse associations between occupational status and weight gain in men and women. Using education as the SES indicator the evidence was slightly less consistent, and when income was used as the SES indicator findings were inconsistent [9]. Jones-Smith et al. completed a cross-national comparison of time trends in overweight inequality by SES among women using repeated cross-sectional surveys from 37 developing countries in the time period from 1989 to 2007. In two out of three studies, higher SES was associated with higher gains in overweight prevalence, in one third of the countries lower SES was associated with higher gains in overweight prevalence. They also observed that increasing wealth was positively related to faster increase in overweight [10].

In a study of American adults, Zhang and Wang found that the association between SES and obesity weakened over three decades, during a time when the prevalence of obesity increased dramatically [11]. In Europe, Charafeddine et al. found a large increase in socioeconomic inequalities in obesity in men but not in women between 1997 and 2004 in Belgium using interview data [12]. Peltonen et al. reported that during the years 1986 to 1994 Body Mass Index (BMI) increased, most markedly in men with university and in women with a secondary school education in the Northern Sweden MONICA Study [13]. Eek and Östergren found increasing socioeconomic inequalities in obesity among young women from 2000 to 2005 in Sweden [14]. In Germany the obesity prevalence increased only moderately between 1990–1992 and 1998, opposing a small reduction in the social gradient in obesity [15].

Distribution and changes in prevalence of obesity in populations may have several explanations. Within populations obesity is suggested to spread in social networks [16]. Unhealthy nutrition and physical inactivity might follow *diffusion patterns* consistent with the spreading of innovations [17] or other health related behaviours [18] like smoking [19]. In the first stage of a behavioural diffusion, the new habit is most prevalent in higher socio-economic groups, for example due to economic privileges, pleasure or fashion. In stage two, the habit becomes more prevalent in all socio-economic groups. Rates among women also rise but lags behind that of men. In the third stage, women reach their peak while prevalence rates start to decline among men - especially in groups with high socioeconomic status. In stage four prevalence rates continues to fall, but at the same time socio-economic inequalities increase [20-23]. The pattern of socioeconomic inequalities in smoking in Europe might mirror such trends, with a steep inverse gradient between education and smoking in affluent North-European countries like Norway and still an opposite gradient in less affluent South-European countries like Portugal lagging behind in socioeconomic development [20].

A geographical perspective on the trends in obesity prevalence might thus be a valuable supplement to the analyses regarding social diffusion [6,24]. The prevalence of obesity might follow a geographical dimension [17,18], starting in affluent areas – ending up highly prevalent in more deprived rural areas [25]. If this causes geographical differences in obesity, it will be due to contextual mechanisms, caused by characteristics of the place. In addition, compositional effects may play a role for example by selective depopulation of people with high socio-economic status in deprived areas [26].

In order to develop effective preventive strategies, knowledge of trends in socioeconomic and geographical differences in risk factor levels is important. Studies following trends over decades in a population with high quality data are also of great interest. Thus, the aims of this study was to examine trends in socioeconomic inequalities in obesity stratified by gender, and to study geographical diffusion patterns in a total Norwegian county population over three decades, from the Nord-Trøndelag Health Study (HUNT) in 1984–86 (HUNT1), through 1995-97 (HUNT2) to 2006–08 (HUNT3).

## Methods

The HUNT Study is a Norwegian population-based general health study consisting of three separate total adult population surveys, HUNT1 in 1984–86, HUNT2 in 1995–97 and HUNT3 in 2006–08 [27]. Every citizen residing in the county of Nord-Trøndelag aged 20 years and above (86,404 in HUNT1; 93,898 in HUNT2 and 93,860 in HUNT3) were invited to participate in the HUNT Study. Overall response rate for the three surveys ranged from 89% (HUNT1) to 54% (HUNT3). Data were collected from questionnaires, blood and urine samples and clinical measurements. To maintain comparability across all three surveys, we limited our analyses to respondents aged 30 through 69 years who had complete data on level of education and BMI. We excluded 18.7% (n = 9664) from HUNT1 (mainly due to missing on self-reported level of education), 1.6% (n = 705) from HUNT2 and 1.1% (n = 435) from HUNT3. The final three study surveys consisted of 42,162 participants in HUNT1, 44,695 in HUNT2 and 40,615 in HUNT3.

### Obesity, body mass index

Height and weight were measured with the participants without shoes wearing light clothes [2]. Height was measured to the nearest centimetre (cm) and weight to the nearest half kilogram (kg). Body Mass Index (BMI) was calculated as body weight in kilograms divided by the squared value of body height in meters (kg/m^2^). According to WHO classifications obesity was defined as ≥30 kg/m^2^. Extreme obesity was defined as BMI ≥ 35 kg/m^2^.

### Education as measure of socioeconomic status

Education was used as a proxy measure of socioeconomic status, measured as the highest level of education completed. In HUNT1 (1984–1986) self-reported data on level of education was used. For HUNT2 (1995–1997) and HUNT3 (2006–2008), we had access to census data from Statistics Norway, resulting in a more complete response rate. The Norwegian Standard Classification of Education was coded by highest completed educational programme attained, and was allocated to the most widely used classification, International Standard Classification of Education (ISECD-97) [28]. In HUNT2 data on highest achieved education was based on the 1995 census, and in HUNT3 on the 2007 census. The seven levels of ISECD-97 were collapsed into three main levels: primary (primary and lower secondary school), secondary (upper secondary and post-secondary school), and tertiary (first and second stage of tertiary education). The general level of education increased considerably in the study period. In the 1980’ies 14% and 9% of men and women had college or university education, compared to 24% and 31% in 2006–08 (Table 1).

**Table 1:** Distribution of the material in three educational levels in The Nord-Trøndelag Health Study (HUNT1, HUNT2 and HUNT3)

	**HUNT1 (1984-86)**				**HUNT2 (1995-97)**				**HUNT3 (2006-08)**			
	**Men**		**Women**		**Men**		**Women**		**Men**		**Women**	
**Education level**	**Number**	**(%)**	**Number**	**(%)**	**Number**	**(%)**	**Number**	**(%)**	**Number**	**(%)**	**Number**	**(%)**
Tertiary	2830	14	1840	9	3914	18	4389	19	4099	24	6276	31
Secondary	6544	31	5828	27	13064	61	13339	57	10320	60	10086	50
Primary	11489	55	13632	64	4399	21	5590	24	2878	17	3929	19
Total classified population	20863	100	21300	100	21377	100	23318	100	17297	101	20291	100

Men and women 30–69 years old.

### Geographical dimensions

**Figure 1:**
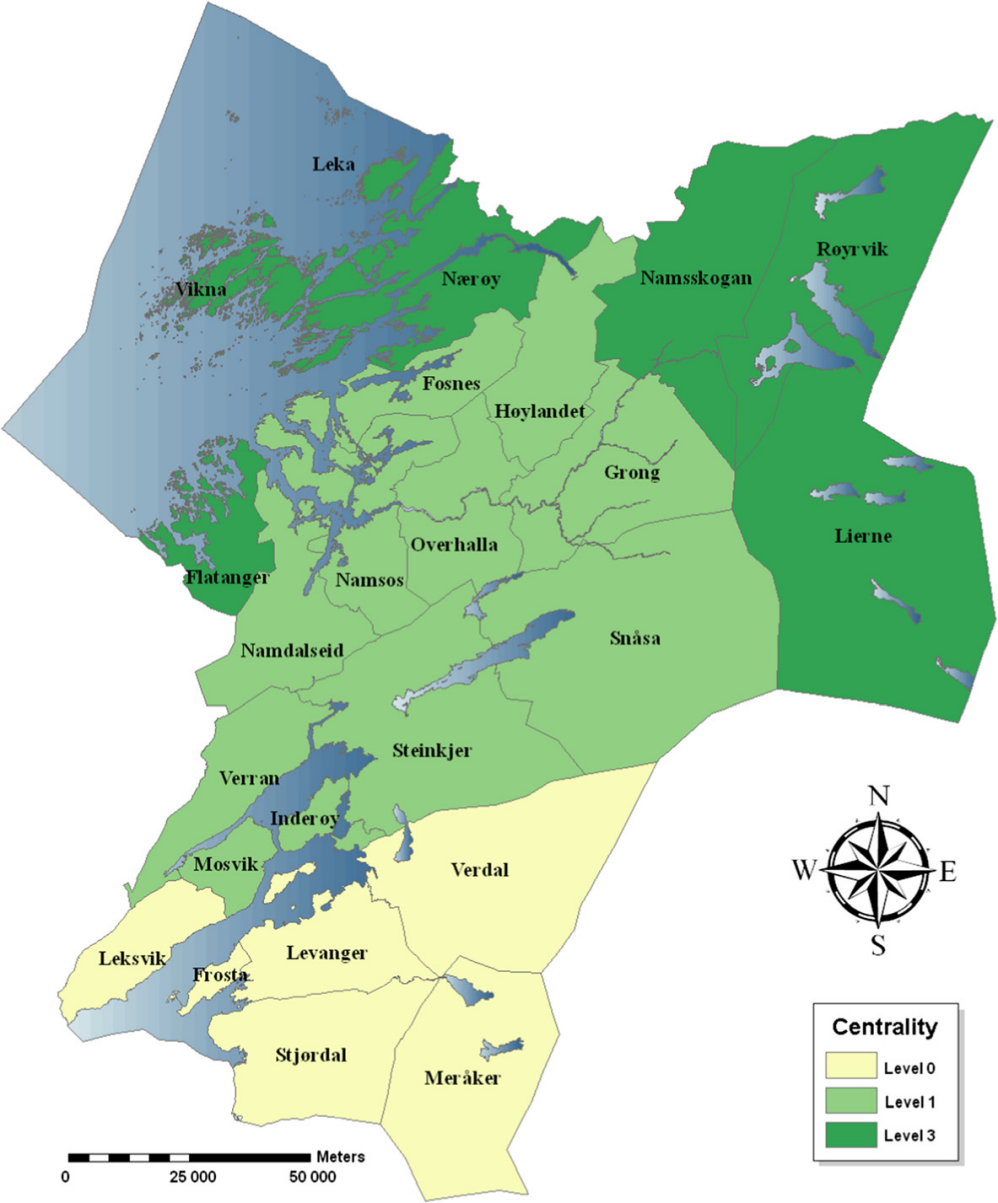
**The Norwegian Centrality Index 2008 applied on the 24 municipalities in the Nord-Trøndelag County, Norway.** Level 0 corresponds to central municipalities, level 3 municipalities are the most distal. (No municipality was at level 2). An international airport located in Stjørdal and the larger town of Trondheim located south of the county, were two aspects influencing the index.

Nord-Trøndelag County is located in the middle of Norway at latitude of 64 degrees north, and was divided into 24 administrative municipalities. The county is mostly rural and sparsely populated; the largest of six small towns has a population of 21,000, the most sparsely populated municipality has a population of 600. Several indicators might be used to capture the two essential dimensions ranging the municipalities in the county, a centrality dimension, and a socio-economic dimension based on mean level of education, income, employment and population stability [26]. In this study we analysed data utilizing a Norwegian Centrality Index from Statistics Norway [29] (Figure 1). Centrality is here defined as the geographical localisation of the municipality in relation to densely populated areas with services like educational institutions, transport nodes, government and other organisational offices etc.

### Statistical analyses

Most analyses were stratified by gender. Age standardised percentages of obesity were calculated using 5 years age groups, the standard population being women and men 30–69 years old as of January 1999 in the Nord-Trøndelag County. To describe differences in prevalence rates of obesity and extreme obesity between the highest and the lowest level of education, both relative and absolute measures were used. Relative differences were calculated by dividing the prevalence rate of obesity in the higher educated group with that of the lower one, absolute differences were calculated by subtracting the prevalence rate of obesity in the higher educated group from that of the lower one.

Relative differences in prevalence rates of obesity (prevalence odds ratios) with 95% confidence intervals were estimated by logistic regression analyses with correction for differences in age structure by including 5-year age groups in the regression model. Highest level of education was always used as reference category. The effect of education on prevalence of obesity was summarised using the Relative Index of Inequality (RII) [30]. RII is recommended when making comparisons over time or across populations [20]. RII transforms level of education, a categorical variable, into a summary measure scaled from zero (top level education) to one (lowest level education) which is weighted to reflect the share of the sample at each level of education.

Absolute and relative differences between central and distal municipalities were calculated between education groups. In order to study associations between centrality and obesity while simultaneously accounting for socio-demographic composition in the municipalities, multilevel statistical models were fitted [31]. Specifically, a two-level binary logistic model based on a logit-link function with Penalized Quasi Likelihood (PQL) approximation and a second-order Taylor Linearization Procedure were used. Models were fitted with the MLwiN programme version 2.26 [32] and fixed effects are reported as Odds Ratios (OR) with 95% confidence intervals (95% CI).

The data were otherwise analysed using the Statistical Package for the Social Sciences (SPSS), version 15.0 for Windows (SPSS Inc., Chicago, IL).

### Ethics

The participation in the HUNT Study was voluntary and based on informed consent. The Norwegian Data Inspectorate approved the HUNT Study. The Regional Committee for Ethics in Medical Research in Mid Norway and the Norwegian Directorate of Health approved this research project and the HUNT2 and HUNT3 surveys. The Regional Committee for Ethics was not yet established when the HUNT1 survey was performed.

## Results

The age adjusted prevalences of obesity and extreme obesity increased successively in all educational groups during the study period. In HUNT1 obesity and extreme obesity was most prevalent among women. In HUNT3, obesity was approximately equally prevalent among men and women (Table 2) whereas extreme obesity still was most prevalent among women. The obesity prevalence ranged from 5.0% in men with high education in HUNT1 to 30.4% in men with low education in HUNT3. A corresponding pattern was not found for BMI above 35; the highest prevalence was found among women with low education in all three surveys reaching 10.3% in HUNT3. The relative inequalities for obesity measured as prevalence ratios were fairly stable in HUNT1, HUNT2 and HUNT3, ranging from 1.6 to 1.9. For extreme obesity the data suggested a reduction in the prevalence ratio from HUNT1 to HUNT2 and a new increase from HUNT2 to HUNT3. The prevalence differences in obesity between people with primary and tertiary education increased from 4.5% and 6.2% among men and women in HUNT1, to 14.0% and 10.6% in HUNT3.

**Table 2:** **Age adjusted prevalence**^**a**^**, prevalence differences and prevalence ratio in educational inequalities in BMI ≥30 kg/m**^**2 **^**and BMI ≥35 kg/m**^**2**^

	**HUNT1 (1984-86)**			**HUNT2 (1995-97)**			**HUNT3 (2006-08)**		
	**Prev. (%)**	**Prev. diff. (%)**	**Prev. ratio**	**Prev. (%)**	**Prev. diff. (%)**	**Prev. ratio**	**Prev. (%)**	**Prev. diff. (%)**	**Prev. ratio**
**BMI ≥30 kg/m**^**2**^									
*Men*									
Primary	9.5	4.5	1.9	19.0	7.5	1.7	30.4	14.0	1.9
Secondary	7.0	2.0	1.4	15.4	3.9	1.3	24.1	7.7	1.5
Tertiary	5.0	Ref.	Ref.	11.5	Ref.	Ref.	16.4	Ref.	Ref.
*Women*									
Primary	14.1	6.2	1.8	21.1	9.2	1.8	27.7	10.6	1.6
Secondary	10.3	2.4	1.3	17.7	5.8	1.5	24.3	7.2	1.4
Tertiary	7.9	Ref.	Ref.	11.9	Ref.	Ref.	17.1	Ref.	Ref.
**BMI ≥35 kg/m**^**2**^									
*Men*									
Primary	1.3	0.9	3.3	2.9	1.4	1.9	7.1	5.1	3.6
Secondary	0.6	0.2	1.5	2.2	0.7	1.5	4.2	2.2	2.1
Tertiary	0.4	Ref.	Ref.	1.5	Ref.	Ref.	2.0	Ref.	Ref.
*Women*									
Primary	4.0	2.5	2.7	6.1	3.0	2.0	10.3	5.7	2.2
Secondary	2.4	0.9	1.6	4.5	1.4	1.5	7.2	2.6	1.6
Tertiary	1.5	Ref.	Ref.	3.1	Ref.	Ref.	4.6	Ref.	Ref.

Men and women 30–69 years old in The Nord-Trøndelag Health Study.

^a^Direct adjustment for age using five years age groups, the standard population being men and women 30–69 years old as of 1. January 1999 in The Nord-Trøndelag Health Study.

The relative inequalities estimated with logistic regression and by applying the Relative Index of Inequality (adjusting for changes in the size of educational groups), suggest a slight reduction in the relative socioeconomic inequalities in obesity among women from HUNT1 to HUNT3 (Table 3).

**Table 3:** **Prevalence odds ratio (pOR)**^**a **^**and relative index of inequality (RII)**^**a **^**in BMI ≥ 30 kg/m**^**2 **^**and BMI ≥ 35 kg/m**^**2 **^**by educational level**

	**Men**						**Women**					
	**HUNT1**		**HUNT2**		**HUNT3**		**HUNT1**		**HUNT2**		**HUNT3**	
	**(n=20863)**		**(n=21377)**		**(n=17297)**		**(n=21300)**		**(n=23318)**		**(n=20291)**	
	**pOR**	**95% CI**	**pOR**	**95% CI**	**pOR**	**95% CI**	**pOR**	**95% CI**	**pOR**	**95% CI**	**pOR**	**95% CI**
**BMI ≥ 30 kg/m²**												
Primary	2.13	(1.75-2.61)	1.71	(1.51-1.94)	1.99	(1.77-2.23)	2.27	(1.83-2.82)	2.04	(1.81-2.30)	1.85	(1.68-2.04)
Secondary	1.53	(1.24-1.89)	1.39	(1.25-1.55)	1.53	(1.39-1.67)	1.54	(1.23-1.94)	1.64	(1.47-1.82)	1.53	(1.41-1.66)
Tertiary	Ref.		Ref.		Ref.		Ref.		Ref.		Ref.	
**RII**	2.60	(2.08-3.26)	1.91	(1.64-2.22)	2.36	(2.05-2.71)	2.71	(2.20-3.34)	2.28	(1.97-2.62)	2.30	(2.03-2.62)
**BMI ≥ 35 kg/m²**												
Primary	3.22	(1.72-6.02)	1.72	(1.24-2.34)	3.15	(2.42-4.11)	3.33	(2.06-5.37)	2.05	(1.64-2.55)	2.32	(1.97-2.74)
Secondary	1.33	(0.67-2.62)	1.45	(1.09-1.92)	2.06	(1.63-2.60)	1.89	(1.14-3.17)	1.57	(1.28-1.92)	1.59	(1.38-1.83)
Tertiary	Ref.		Ref.		Ref.		Ref.		Ref.		Ref.	
**RII**	6.94	(3.41-14.15)	1.87	(1.28-2.74)	3.94	(2.90-5.37)	4.21	(2.78-6.38)	3.44	(2.70-4.38)	3.08	(2.48-3.82)

Men and women 30–69 years old in HUNT1 (1984–1986), HUNT2 (1995–1997) and HUNT3 (2006–2008).

^a^Age adjusted pOR and RII by 5-years age groups.

**Figure 2:**
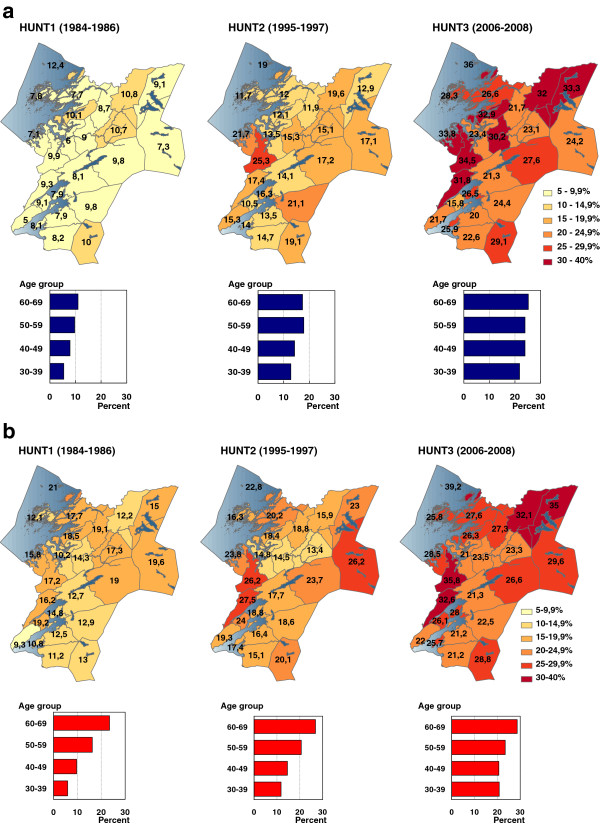
**Age adjusted obesity prevalence (BMI ≥ 30) in 24 municipalities in the Nord-Trøndelag County Norway, (a) men and (b) women in HUNT1 (1984–86), HUNT2 (1995–97) and HUNT3 (2006–08).** The bar charts present age stratified prevalence in obesity in the total material for each survey.

Regarding geographical diffusion, Figure 2 shows the age adjusted prevalence of obesity in the 24 municipalities in the county in HUNT1 (1984–86), HUNT2 (1995–97) and HUNT3 (2006–08), and suggests a diffusion of obesity from central to distal and more rural districts in the period between HUNT2 (1995–97) and HUNT3 (2006–08). Figure 2a and 2b also presents bar charts with age stratified obesity prevalence from each decade. The charts show that the prevalence of obesity has increased relatively more in younger compared to older adults over time.

The data presented in Table 4 quantifies the impression from Figure 2, demonstrating a prevalence ratio between central and distal municipalities increasing from 1.05 to 1.30 and corresponding prevalence differences increasing from 1.69% in HUNT1 to 6.47% in HUNT3. However, the trend was opposite to that in the preceding decade.

**Table 4:** **Age adjusted prevalence**^**a**^**, prevalence difference and prevalence ratio in BMI ≥30 kg/m**^**2 **^**in 24 municipalities ranged by the Norwegian Centrality Index**

	**HUNT1 (1985–97)**			**HUNT2 (1995–97)**			**HUNT3 (2006–08)**		
**Centrality index**	**Prevalence (%)**	**Prevalence diff. (%)**	**Prevalence ratio**	**Prevalence (%)**	**Prevalence diff. (%)**	**Prevalence ratio**	**Prevalence (%)**	**Prevalence diff. (%)**	**Prevalence ratio**
Central	10.22	Ref.	Ref.	16.36	Ref.	Ref.	21.69	Ref.	Ref.
Intermediate	10.91	0.69	1.17	16.77	0.41	1.03	23.18	1.49	1.07
Distal	11.91	1.69	1.17	17.22	0.86	1.05	28.16	6.47	1.30

Men and women 30–69 years old in The Nord-Trøndelag Health Study (HUNT).

^a^Direct adjustment for age using five years age groups, the standard population being men and women 30–69 years old as of 1 January 1999 in The Nord-Trøndelag Health Study.

The geographical pattern might, however, be explained by lower education levels in rural districts and effects of selective mobility patterns. Thus, adjustment for education at individual level was made. Table 5 shows the relative differences in obesity between central, intermediate and distal municipalities adjusted for gender, age and education. Adjustment for education attenuated the estimates for the distal municipalities slightly. Data from the most distally located municipalities showed a significant increased relative prevalence of obesity in the last survey compared to previous surveys.

**Table 5:** **Relative prevalence differences of obesity (BMI ≥30 kg/m**^**2**^**) in municipalities ranged by the Norwegian Centrality Index**

	**HUNT1**				**HUNT2**				**HUNT3**			
	**Model 1**^**a**^		**Model 2**^**b**^		**Model 1**^**a**^		**Model 2**^**b**^		**Model 1**^**a**^		**Model 2**^**b**^	
**Centrality Index**	**OR**	**(95% CI)**	**OR**	**(95% CI)**	**OR**	**(95% CI)**	**OR**	**(95% CI)**	**OR**	**(95% CI)**	**OR**	**(95% CI)**
Central	1	ref.	1	ref.	1	ref	1	ref.	1	ref.	1	ref.
Intermediate	1.20	(1.02-1.42)	1.20	(1.02-1.41)	1.05	(0.85-1.29)	1.05	(0.86-1.28)	1.14	(0.93-1.38)	1.14	(0.95-1.36)
Distal	1.24	(1.02-1.50)	1.20	(0.99-1.45)	1.10	(0.87-1.38)	1.06	(0.84-1.32)	1.40	(1.13-1.75)	1.34	(1.10-1.65)

Men and women 30–69 years old in The Nord-Trøndelag Health Study (HUNT).

Adjusted for age, sex (model 1) and education (model 2), two level analysis.

^a^Adjusted for age and sex.

^b^Model 1 + education.

## Discussion

The prevalence of obesity increased successively in all socioeconomic groups in this Norwegian adult population aged 30 to 69 years old during the study period. In HUNT1 (1984–86) obesity (BMI ≥ 30) was most common among low-educated women (14.1%) and in HUNT3 (2006–08) among low-educated men (30.4%). Relative socioeconomic inequalities in obesity measured by level of education did not show a clear trend. However, the absolute inequalities increased. A geographical diffusion from central to distal districts was observed during the last decade, only partly explained by inequalities in educational levels between central and distal areas.

This unique large population based material with standardised clinical measurements of height and weight, allows for monitoring trends in health related behaviour and subsequent consequences from the 1980ies up to present time. The missing data on education in HUNT1 was due to inclusion of the question of education in a second questionnaire that participants should return by mail after the health examination. We did not have any other available socio-economic measurements on the excluded group. However, individuals excluded due to missing information on education had quite similar characteristics regarding BMI, sex and employment; mean BMI 25,2 kg/m^2^ vs. 25,4 kg/m^2^, proportion of men was 51% vs. 49% and the proportion being employed was 83% vs. 82%. These data did not indicate any serious selection bias by SES. As the attendance declined over time, a comprehensive non-attendance study in HUNT3 was conducted. Among non-participants the prevalences of diabetes mellitus and chronic disorders were higher compared to that reported by participants, whilst the opposite pattern was found for symptoms and illnesses. Registry data demonstrated that the non-participants had lower socioeconomic status and higher mortality than participants [33]. Thus, there is reason to believe that among non-participants there were more people with low socioeconomic status and more people with obesity compared to participants. This non-response pattern has probably been present in the previous surveys as well. However, the participation declined over time, and this may have led to underestimation of the socioeconomic differences in obesity in HUNT3 compared to HUNT1. The number of participants in the smallest municipalities gave some unstable estimates, but the geographical diffusion trend was convincing.

We used education as an indicator of socioeconomic status. Different indicators of socioeconomic status may be differently associated with obesity. The ideal would have been to have access to both occupation and income in addition to education as a measure of SES [9].

Based on local knowledge, the National Centrality Index developed by Statistics Norway may not reflect the counties’ geography in an optimal way (Figure 1). This may have attenuated the geographical differences somewhat compared to what might have been shown with an index based on other principles. However, we decided to use the national index rather than to develop a local variant. The municipalities varied considerably in population size. Thus, an alternative approach for analyses of geographical diffusion would be to use smaller, more equal areas like neighbourhoods.

We observed an increasing prevalence of obesity in all socioeconomic groups in this Norwegian population during the last three decades. This may indicate a diffusion of obesity in stage two and three according to diffusion theory, where the behavior associated with obesity still is increasing in all groups but already has become more prevalent in lower socio-economic groups [17]. However, obesity is probably the result of interplay between several types of behavioural patterns, which together lead to an imbalance between energy intake and consumption. How well the theory of diffusion of innovations is suited to describe such trends, is an unanswered question.

The observed secular trend, with increase in obesity across all socioeconomic groups and geographical locations, might also suggest a more common contextual influence than individual modifying factors. If the theory of social diffusion is applicable, we might in the years to come expect that the increasing prevalence of obesity will first culminate in the highest socioeconomic groups, thus increasing the socioeconomic inequalities, if proper health policy interventions are not undertaken. The ability to cope with negative effects from an environment offering unlimited quantities of cheap high-energy food and low incentives to everyday physical activity, will probably be highest among people with high socioeconomic status. People in this group probably know more about the benefits of physical activity and healthy diet, they have as a group better behavioural skills, a higher level of motivation caused by better life prospects and easier access to healthy food [9]. This means that the necessary preventive initiatives to avoid increasing obesity and attached health problems should be design to influence the total population but with highest impact on lower socioeconomic groups.

The observed gender pattern might also partly be in line with diffusion theory. Men may have been the first to develop obesity in this population in the period before the data was collected. However, women had the highest prevalence of obesity (BMI ≥ 30) in HUNT1, men the highest in HUNT3 [2]. If future development follows the theory, we might in the years to come expect that prevalence rates first start to culminate among men in groups with high socioeconomic status. However, gender equality in modern societies may influence gender patterns of health-related behavior.

The observed diffusion of obesity from central to distal regions between HUNT2 and HUNT3 is in line with the diffusion theory. Although we observed a weak reverse trend in the first decade, the relatively high prevalence of obesity in the most distal municipalities in the last cross-sectional survey was marked. Geographical diffusion progresses both horizontally, for instance from neighbour to neighbour areas, and vertically from urban to rural districts [17]. The geographical diffusion pattern might be a result of different socio-cultural living conditions in urban and rural areas. However, as people in densely populated areas live closer together, the social pressure towards looking slim and joining leisure time physical activities might be higher. The offer of organized sports activities is higher and the travel distances shorter in more centralised areas, thus paradoxically making the car less necessary than in the rural districts, allowing walking or bicycling instead. But other processes might also explain the geographical patterns, like restructuring of society and migration [34]. Comprehensive analyses of these factors were outside the scope of this paper. However, controlling for education in the analyses of geographical variations shown in Table 5, may have adjusted the effects of emigration of well-educated people from municipalities with economic stagnation. The social pattern and geographical diffusion of obesity demonstrated in this study are in any case important for public health surveillance and preventive strategies.

The obesity epidemic might be explained by trends in every day physical activity and dietary factors. Men with low socioeconomic status are more likely to have physically demanding occupations compared to men with high socioeconomic status. The proportion of workers employed in physically demanding activities is decreasing [35]. But leisure physical activity is reported to increase in the last decade, more pronounced in high socioeconomic groups [36] and in central living areas.

Giskes et al. examined socioeconomic inequalities in dietary factors associated with obesity among adults in Europe, demonstrating that the direction of associations between socioeconomic status and energy intake was inconsistent. However, lower socioeconomic groups are probably less likely to consume fruits and vegetables [37]. In another paper from the HUNT Study Nilsen et al. found that higher levels of parents’ and particularly mothers’ education, was associated with healthier dietary habits among adolescents [38].

Associations between socioeconomic status and obesity are consistent in developed economies. However, associations between health related behaviours and obesity are often inconsistent. This might first of all reflect difficulties in assessing physical activity and dietary factors. Nevertheless, our data underscore a view of obesity as a social phenomenon, for which appropriate action includes targeting social, cultural and economic factors [8]. For public health policy, the increasing prevalence and the socioeconomic and geographical inequalities in obesity are huge challenges.

## Conclusions

The prevalence of obesity increased in all socioeconomic groups in this Norwegian adult county population from the 1980ies up to present time. The data did not suggest increasing relative inequalities, but increasing absolute socioeconomic differences and a geographical diffusion towards rural districts. Public health preventive strategies should be oriented to counteract the obesity epidemic in the population, with particular emphasis on counteracting the high prevalence in lower socioeconomic groups and in rural districts.

## Competing interests

The authors declare that they have no competing interests.

## Authors’ contributions

SK have made substantial contributions to conception and design, acquisition of data, interpretation of data, have led drafting of the manuscript, revising it critically for important intellectual content and given final approval of the version to be published. LE, ERS and HT have made substantial contributions to conception and design, analysis and interpretation of data, revising the manuscript critically for important intellectual content and given final approval of the version to be published. JHB and SW have made substantial contributions to conception and design, have been involved in drafting the manuscript and revising it critically for important intellectual content, and given final approval of the version to be published. AL, KM, TLH and JH have made substantial contributions to conception and design, acquisition of data, revising the manuscript critically for important intellectual content and given final approval of the version to be published.

## Pre-publication history

The pre-publication history for this paper can be accessed here:

http://www.biomedcentral.com/1471-2458/13/973/prepub
